# Clinical features and treatment of vulvar Merkel cell carcinoma: a systematic review

**DOI:** 10.1186/s40661-017-0037-x

**Published:** 2017-01-25

**Authors:** Austin Huy Nguyen, Ahmed I. Tahseen, Adam M. Vaudreuil, Gabriel C. Caponetti, Christopher J. Huerter

**Affiliations:** 1 Creighton University School of Medicine, 2500 California Plaza, Omaha, NE 68102 USA; 2 Department of Pathology and Laboratory Medicine, Perelman School of Medicine, University of Pennsylvania, 3400 Spruce Street, Philadelphia, PA USA; 3 Division of Dermatology, Creighton University School of Medicine, 2500 California Plaza, Omaha, NE 68102 USA

**Keywords:** Vulvar neoplasms, Skin neoplasms, Merkel tumor, Neuroendocrine tumors

## Abstract

**Background:**

Merkel cell carcinoma is a rare and aggressive neoplasm originating from mechanoreceptor Merkel cells of the stratum basale of the epidermis. Cases affecting the vulva are exceedingly rare, with the currently available literature primarily in case report form.

**Body:**

Systematic review of the PubMed database returned 17 cases of Merkel cell carcinoma affecting the vulva. Patients presented at a mean age of 59.6 years with a firm, mobile vulvar mass. Symptoms of pain, erythema, pruritus, edema, and ulceration have been reported. Tumor histology is consistent with that of neuroendocrine tumors and typical Merkel cell carcinomas. Neuroendocrine and cytokeratin immunostains are frequently utilized in histopathological workup. Surgical management was the unanimous first-line therapy with adjuvant radiation in most cases. Recurrence occurred in 70.6% of patients at a mean follow-up of 6.3 months. Mortality was at 47.0% at a mean of 7.8 months after initial operation.

**Conclusion:**

Merkel cell carcinoma affecting the vulva is an extremely rare and highly aggressive neoplasm. The present review of published cases serves to comprehensively describe the clinical course and treatment approaches for vulvar Merkel cell carcinoma.

## Background

Merkel cell carcinoma (MCC) is a rare and aggressive neoplasm first described in 1972 by Toker [1]. The tumor is thought to originate from the Merkel cell mechanoreceptors located in the stratum basale of the epidermis [2]. Although rare, the incidence of this neoplasm is increasing due to the advancing age of the population, higher rates of sun exposure, and a growing proportion of immunocompromised individuals [2]. MCC occurs predominately in the elderly with an average age of onset at 69 years old and a slightly higher prevalence in males (1.56:1 Male:Female) [3]. Additional risk factors include Caucasian race (incidence of 0.23 per 100,000) [2] and immunosuppression, with a younger age at presentation for immunocompromised individuals [4]. The neoplasm is predominately found in the head and neck (41–50%), followed by the extremities (32–38%), and then the trunk (12–14%) [2]. Regarding the etiology of the tumor, a recent study [5] described a polyomavirus detected in 43 to 100% of MCC tissue samples. The pathogenesis of this Merkel cell polyomavirus, however, still requires further investigation.

The primary lesion of MCC typically presents as a solitary, painless, rapidly growing, red to bluish nodule [2, 6]. Definitive diagnosis requires histopathologic analysis of a biopsy. Upon hematoxylin and eosin staining, the lesion will appear similar to other neuroendocrine tumors consisting of small round cells, hyperchromic nuclei, frequent mitosis, and variable architecture [2]. With hematoxylin and eosin staining alone it is difficult to differentiate MCC from other small cell tumors, especially metastatic small cell cancer of the lung. Accordingly, immunohistochemical evaluation is recommended [2, 7]. An immunopanel including cytokeratin 20 (CK20) and thyroid transcription factor-1 (TTF-1) allows the greatest sensitivity and specificity for excluding small cell lung cancer [7]. CK20 is highly sensitive for MCC (positive in 89 to 100% of cases) while TTF-1 is sensitive for small cell lung cancer (positive in 83 to 100% of cases), and consistently negative in MCC [7].

While various staging guidelines have been proposed historically, the most recent and widely accepted staging guideline is the AJCC staging system [7, 8], which draws upon evidence from the analysis of 5823 cases in the National Cancer Database with a median follow-up of 64 months [3, 7]. Staging affects an individual’s prognosis, with 5-year survivals rates of 79% at stage IA to only 18% at stage IV [3]. Additionally, 50 to 70% of patients will develop lymph node metastases and 33 to 70% of those will go on to develop distant disease [2]. The most common sites of metastasis are as follows: brain (18%), liver (13%), lung (10–23%), bone (10–15%), distant skin (9–30%), and distant lymph node (9%) [2]. Due to this high rate of metastasis, patients with a primary MCC should be screened for nodal metastases with sentinel lymph node biopsy. Additionally, other imaging modalities are gaining importance during diagnostic workup. For example, PET/CT may be useful in identifying distant metastases [7]. In one article reviewing 102 patients, PET/CT altered the stage and treatment course in 22% of the cases [9].

Treatment of MCC varies by stage, with the main categories being treatment of the primary lesion, treatment of regional disease, and treatment of distant metastasis. Surgical excision is the treatment of choice for primary lesions [2, 6, 7]. The two surgical approaches are wide local excision with 1 to 2 cm margins and depth to the investing fascia or Mohs surgery. These approaches have equal efficacy if they attain tumor-free margins [2]. In addition to surgery, adjuvant radiotherapy is often recommended. Postoperative radiation has shown to lower the risk of local and regional recurrences and has been associated with a longer overall survival [7]. In the case of a positive node, adjuvant therapy to the nodal basin is recommended and associated with longer disease-free survival [2, 6]. Adjuvant therapy often consists of surgical removal of the basin nodes or regional radiotherapy, or a combination of the two. It is recommended to get a multidisciplinary tumor board consultation in metastatic disease, and to consider any combination of additional surgery, radiotherapy, and chemotherapy [7]. Recommendations concerning follow up for patients after MCC treatment are broad [7]. This allows for individualization based on patient factors and physician preference. The standard regimen is routine physical and skin exam every 3 to 6 months for the first 2 years, followed by every 6 to 12 months thereafter. This recommendation takes into consideration that the median time to recurrence is 8 months with 90% of recurrences happening within 2 years [7].

While MCC is rare, a primary lesion affecting the vulva is extremely rare. The vulvar location of primary tumors is especially unique as cutaneous MCC is characteristically more frequent in men [3]. A study of 3870 MCC cases from the National Cancer Institute’s Surveillance, Epidemiology, and End Results Program database found only two cases (0.05%) affecting the vulva [10]. Currently, all data on vulvar MCC is found in “case report and literature review” form. The present study seeks to comprehensively review the available patient data to accurately describe the clinical course and treatment approaches for vulvar MCC.

## Main text

### Search strategy

The National Library of Medicine’s PubMed database was systematically searched to December 2016 without date restrictions using the following search terms: “vulva” and “vulvar” combined with “Merkel cell carcinoma,” “cutaneous apudoma,” “neuroendocrine carcinoma,” “trabecular carcinoma.” Titles and abstracts were screened for possible inclusion, followed by full text of potentially relevant studies. Included studies were original studies discussing the clinical course (including presentation, diagnostic workup, treatment, and outcome) of patients with MCC affecting the vulva. Studies were excluded if not written in English, not of primary human subjects, or not malignancies of the vulva.

**Fig. 1:**
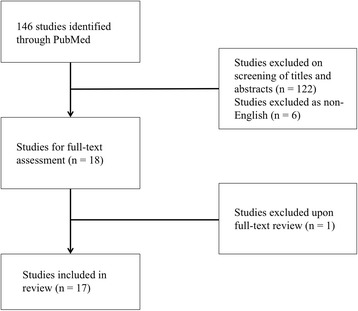
Initial PubMed search returned 146 studies. Screening by title and abstract left 18 studies, of which full text was reviewed. Ultimately, 17 cases were included in this review

Initial PubMed search (see Fig. 1) returned 146 potentially relevant articles. After screening of titles and abstracts, the full text of 18 studies was retrieved for review [11–27]. Upon full text review, one study was excluded for providing insufficient clinical data on patient-level clinical course (i.e. this study was a large cancer database study of general MCC with minimal summary statistics provided specifically for vulvar MCC). Ultimately, 17 case reports were included in this review. The greatest number of cases were reported in the United States (7 cases), followed by Spain (2 cases).

### Clinical presentation

Patients presented at a mean age of 59.6 years (range 28–79 years). The clinical presentation of the 17 included cases are summarized in Table 1. Lesions were most commonly located on the labia majora (*n* = 9, 52.9%) with no distinct predilection for side (left, *n* = 9; right: *n* = 7; unreported: *n* = 1). 23.5% of cases (*n* = 4) extended to affect the vaginal wall, while other affected anatomical locations included the labia minora, paraclitoral or the bartholin gland. Patients generally complained of a rapidly growing mass (average history of 4.7 months, range of 1–18 months) that was 7.5 cm (range 1.75–47.5 cm) on average at presentation. The lesions was described as a firm, painless (*n* = 3, 17.6%) or tender (*n* = 5, 29.4%) nodule that was mobile. Cases reported associated pruritus (*n* = 2, 11.8%), swelling or edema (*n* = 3, 17.6%), ulceration (*n* = 4, 23.5%), and erythema (*n* = 2, 11.8%). Bleeding and purulent discharge was reported in a fraction of cases (each, *n* = 2, 11.8%). Discoloration (*n* = 3, 17.6%) was reported as yellow, purple or brown.

**Table 1:** Clinical presentation of vulvar merkel cell carcinoma (*n* = 17)

Characteristic	n (% or range)
Mean age (years)	59.6 (28–79)
Mean tumor diameter (cm)	7.5 (1.8–47.5)
Mean disease duration (months)	4.7 (1–18)
Location^a^	
Labia majora	9 (52.9)
Labia minora	3 (17.6)
Paraclitoral	1 (5.9)
Bartholin gland	3 (17.6)
Intravaginal extension	4 (23.5)
Inguinal	1 (5.9)
Vulva, Unspecified	1 (5.9)
Clinical findings^a^	
Firm	2 (11.8)
Painless	3 (17.6)
Tender	5 (29.4)
Mobile	2 (11.8)
Pruritus	2 (11.8)
Swelling/edema	3 (17.6)
Ulceration	4 (23.5)
Erythema	2 (11.8)

^a^Sum exceeds 100% due to non-mutually exclusive categories

### Workup

**Fig. 2:**
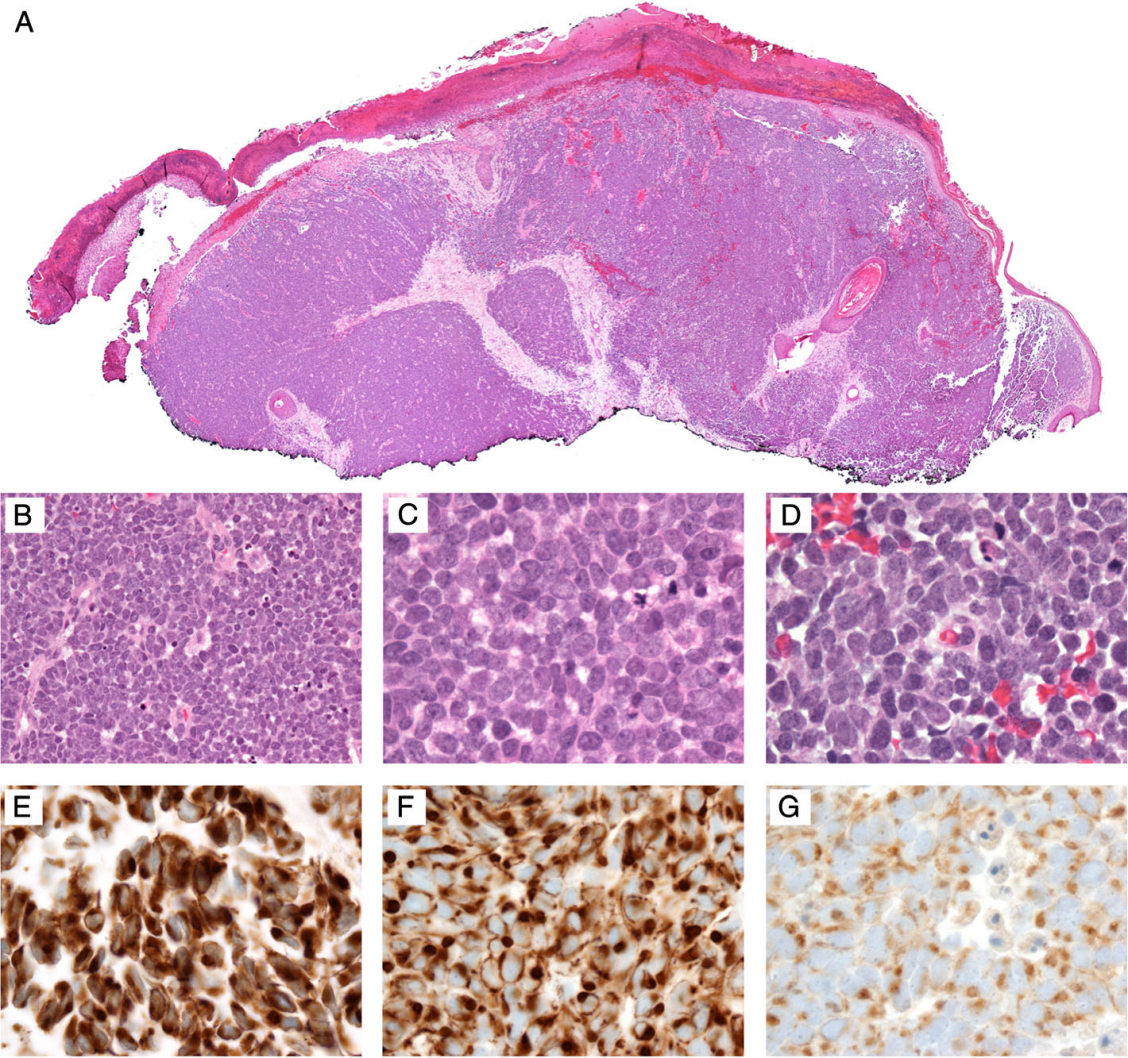
Photomicrographs of a typical Merkel cell carcinoma at **a** 4x, **b** 40x, and **c**–**d** 100x objectives. Hematoxylin and eosin staining demonstrates small, undifferentiated cells with high N/C ratio and scanty cytoplasm. Typical immunopanel demonstrates positive staining with **e** cytokeratin AE1/AE3 (100x oil immersion), **f** CK 20 (100x oil immersion), and neuroendocrine markers such as **g** chromogranin (100x oil immersion)

Blood and urine chemistries were unremarkable in the few cases reporting values, excepting occasional comorbidities that did not impact vulvar MCC diagnostics. Histopathological evaluation (Table 2) was the primary diagnostic modality, performed using needle biopsy (*n* = 5, 29.4%), incisional or excisional biopsy (*n* = 5, 29.4%), evaluation following tumor resection (*n* = 1, 5.9%), or unspecified (*n* = 6, 35.3%). Histologically, vulvar MCC is typical of neuroendocrine tumors and traditional MCC (see Fig. 2). Routine evaluation with hematoxylin and eosin demonstrated small, undifferentiated, hyperchromatic cells with a high N/C ratio and scanty cytoplasm. Cells were arranged in nested, trabecular pattern (*n* = 11, 64.7%) separated by fibrous connective bands and/or were in sheets (*n* = 3, 17.6%). Indicators of aggressive malignancy were common, including high mitotic index (*n* = 8, 47.1%), irregular nuclei (*n* = 4, 23.5%), necrotic and apoptotic cells (*n* = 6 and 4, respectively), hemorrhage (*n* = 2, 11.8%) and ulcerated dermis (*n* = 2, 11.8%). Electron microscopy was reported in 7 cases (41.2%). In these cases, tumor cells exhibited cytoplasmic membrane-bound dense core neurosecretory granules (*n* = 6, 85,7%) and intermediate filaments (*N* = 5, 71.4%).

**Table 2:** Histopathological evaluation of vulvar merkel cell carcinomas

Characteristic	n (%)
Histologic finding (*n* = 17)	
Small cells	12 (70.6)
High N/C ratio, scant cytoplasm	12 (70.6)
Nests, islands, trabecular	11 (64.7)
Hyperchromatic	10 (58.8)
High mitotic index	8 (47.1)
Necrosis	6 (35.3)
Irregular nuclei	4 (23.5)
Fibrous	4 (23.5)
Apoptosis	4 (23.5)
Sheets	3 (17.6)
Hemorrhage	2 (11.8)
Ulceration	2 (11.8)
Electron microscopy (*n* = 7)^a^	
Dense core granules	6 (85.7)
Intermediate filaments	5 (71.4)
Immunostaining (*n* = 14)^a^	
Neuroendocrine markers	
Chromogranin	7 (50)
NSE	7 (50)
Synaptophysin	6 (42.9)
PGP 9.5	2 (14.3)
Keratin stains (*n* = 13)^a^	
Pancytokeratin AE1/AE3	7 (53.8)
CAM5.2	4 (30.8)
Low molecular weight CK	3 (23.1)
CK7	1 (7.7)
CK8	2 (15.4)
CK18	3 (23.1)
CK19	1 (7.7)
CK20	4 (30.8)
Perinuclear dot/granular	7 (53.8)

*Abbreviations*: *CK* cytokeratin, *N/C ratio* nuclear/cytoplasmic ratio, *NSE* neuron specific enolase, *PGP* protein gene product

^a^Total n, reflected in percentages, is less than 17 due to inconsistent reporting of electron microscopy or positive and negative immunostains

Immunostain results were reported in all but 2 cases and are summarized in Table 2. Neuroendocrine and keratin stains were the most commonly used for histopathological diagnostic workup. Cases commonly stained positive for neuron specific enolase (*n* = 7), chromogranin (*n* = 7), and synaptophysin (*n* = 6). Keratin stains included pancytokeratin AE1/AE3 (*n* = 7), CAM5.2 (*n* = 4), and low molecular weight cytokeratins (*n* = 3). Generally, cytokeratin immunoreactivity patterns demonstrated perinuclear dots and/or cytoplasmic granularity. Other immunostains with two or fewer positives included CD56, Ki-67, endomysial antibody, carcinoembryonic antigen, and S100. Stains with no positives included CD45, TTF-1, HMB45, desmin, vimentin, smooth muscle actin, CA125, CD31, and CD34.

Ultrasound was performed in 4 cases. While three reports demonstrated no tumor findings on ultrasound, one case [11] reported ultrasound to detect a well circumscribed, heterogeneous, cystic mass with irregular vascularity. Plain chest radiographs were unremarkable in all 9 cases reporting use of X-ray imaging, except one case [20] in which extensive lung metastases were shown. CT scans, performed in 10 cases, appeared to be the most sensitive for detection of metastases.

### Management and outcome

All patients received surgical excision as first line therapy. Vulvectomy was performed in ten patients (58.8%). Wide local excision was performed in 4 cases (23.5%) with 2 cm margins, where reported. Excision was unable to be completed in one case due to inaccessibility of the lesion, and surgical approach was not reported in another case until recurrence. Inguinal lymph node dissection was reported in 10 cases (58.8%). Some form of adjuvant radiotherapy was administered in 11 cases (64.7%). Of those reporting sufficient data, radiation dosage was 400 to 6500 cGy at first dose of adjuvant radiotherapy, with additional courses at varied doses. Radiation was administered locally in the pelvic region, with some cases administering radiation at inguinal or even para-aortic lymph nodes. 11 cases reported radiation as a part of the treatment regimen, however three did not provide follow-up results for the patient, as radiotherapy had not been performed at the time the cases were written [11, 12, 15]. Of the eight patients with reported follow up, six patients experienced recurrence at an average of 5.8 months after treatment [14, 17, 21, 24–26]. Five of these cases reported the amount of radiation therapy, with an average of 6008 cGy [14, 21, 24–26]. Six patients had recurrent disease after radiation therapy, three died after 0 [14], 3 [25], and 4 months [26] post-radiotherapy. Three patients with recurrence were still alive at 0 [24], 0 [21], and 8 months [17] post-radiotherapy. Of the two patients who did not experience recurrence, one patient received 5940 cGy and died at 8 months post-radiotherapy due to sepsis [18], while the other received 5000 cGy plus an additional 5000 cGy targeted at original mass location and was still alive at 24 months post-radiotherapy [16].

Patient prognosis was poor. Recurrence occurred in 11 patients (64.7%) at a mean follow-up of 4.7 months (range 2–9 months). Two patients were disease-free at 13 and 24 months follow-up, respectively (three patients lost to follow-up or outcome not reported). Recurrent lesions were managed surgically or with cisplatin and etoposide combination chemotherapy (*n* = 5; 2 cases did not specify regimen). Eight patients (47.0%) succumbed to advanced disease, with death at an average of 9.6 months after initial surgical operation (range 0.36–20 months post-operation). The clinical course of all included cases is summarized in Table 3.

**Table 3:** Summary of Clinical Presentation, Treatment, and Outcome of Vulvar Merkel Cell Carcinoma Cases

Case	Age	Location/Size	Presentation	Treatment	Outcome + Survival
Bottles et al. 1984 [27]	73	Left labia majora.	Minute ulcer w/chronic ulceration	Initial: Testosterone + hydrocortisone cream to heal initial ulcer. 10 months, 3 weeks: Vulvectomy + Left Inguinal lymphadenectomy	9 Months: Local raised, nodular, erythematous tumor 3 x 2 cm + Left Inguinal LN metastases 11 months (11 days post operation): death due to acute MI + cardiopulmonary failure. Inguinal and paraaortic nodes, bone, liver, pulmonary vessel metastases.
Copeland et al. 1985 [26]	59	Left labium majus 6 x 8 cm	18 month history of painful lump + Local tumor + Left Inguinal LN metastases	Initial: Left hemivulvectomy + lymphadenectomy + Radiotherapy 8 months: Vulvar lesion excision.	8 months: Vulvar + several pulmonary metastases. 12 months: Death
Husseinzadeh et al. 1988 [25]	47	Right labium majus + vaginal introit. 4.2 x 3 cm	3 month history of right labial/groin swelling with brown vaginal discharge and pain on sitting. Local tumor + bilateral inguinal LN metastases	Initial: Vulvectomy + Bilateral lymphadenectomy + Radiotherapy 3 months: Excision + Chemotherapy	3 months: right thigh nodule, forehead nodule, single nodular lesion in left hilar region. 6 months: Death. Autopsy: hilar, lung, liver, pancreas metastases.
Chandeying et al. 1989 [24]	28	Right labium majus 4 cm	1 month history of painless lump. Local tumor + bilateral inguinal LN metastases	Initial: Vulvectomy + bilateral lymphadenectomy + radiotherapy	3 months: Right leg pain improved with symptomatic treatment. 4 months out: Alive. No subsequent follow up.
Loret de Mola et al. 1993 [23]	28	Left fourchette 1.5 x 2 cm	3 month history of Vulvar growth and irritation. Local tumor	Initial: local excision. 2 months: Wide local excision + left inguinal lymphadenectomy 8 months: chemotherapy	8 months: liver metastases. 20 months: Death.
Chen 1994 [22]	68	Left paraclitoral 3 x 2.5 cm	1 month history of mass. Local tumor.	Initial: Local excision 10 months: Chemotherapy.	9 months: bilateral Inguinal LN and liver metastases. 10 months = Vulva, scalp, bone and paraaortic LN. 17 months: Death.
Scurry et al. 1996 [21]	68	Left labium minus + fourchette 4 x 3 cm.	5 month history of painless lump with rapid growth in last 2 weeks. Local tumor + overlying discolored purplish skin. bilateral inguinal LN metastases	Initial: Vulvectomy + bilateral inguinal and Left pelvic Lymphadenectomy 2 months: Radiotherapy	Residual pelvic nodes post treatment. 2 months: para aortic LN. 5 months: Alive with residual disease.
Gil et al. 1997 [19]	74	Right labium majus 9 cm	3–4 month history of local tumor	Initial: Wide Local excision	13 months: free of disease
Fawzi et al. 1997 [20]	78	Right vulvar mass 5.5 x 4 cm	1 month history of perineal itching and discomfort. Pulmonary LN metastases.	Initial: Radical vulvectomy + bilateral inguinal LN dissection	20 days postoperative: break down of right groin site and subsequent death due to bleeding. No autopsy.
Hierro et al. 2000 [18]	79	Left labium minus 2.5 cm	Local tumor	Initial: local excision. 2 months: Radiotherapy	2 months local recurrence and regional LN metastases. 10 months: Death
Nuciforo et al. 2004 [17]	62	Right labia majora 20 mm	Local painful tumor.	Initial: local excision. 3 months: Radical vulvectomy + Radiotherapy.	3 months: bilateral inguinal LN metastases. 11 months: abdominal and mediastinal LN. 19 months: Alive with Several abdominal and thoracic metastases.
Khoury et al. 2005 [16]	49	Right vulvar mass 2 cm	Spontaneously ruptured Bartholin’s gland abscess with small induration at the site.	Initial: Drained abscess + wide local excision + bilateral LN dissection + Radiation therapy	24 months: Alive with no evidence of recurrence.
Pawar et al. 2005 [15]	35	Left labium majus 4 x 6 cm	One week history of painful swelling of the vulva + purulent discharge + LN mass	Initial: Drained abscess + antibiotics + partial excision	No follow up, patient planned to receive radiotherapy in her home country.
Mohit et al. 2009 [14]	50	Left labia majora 3–4 cm	3 month history of palpable mass.	Initial: local excision 2 months: Radiotherapy 2 months, 3 weeks: radical vulvectomy 9 months: Chemotherapy	2 months: Recurrent mass 10 x 12 cm w/spontaneously bleeding ulcerations 9 months: left hip pain 10 months: no evidence of metastases 11 months: death due to Pulmonary embolism secondary to DVT of LLE.
Sheikh et al. 2010 [13]	63	Right labium majus 5 x 7 cm	Post menopausal bleeding with fungating primary lesion.	Initial: wide local excision.	2 months: local + distant recurrence with multiple firm inguinal LN bilaterally + death before follow up treatment
Iavazzo et al. 2011 [12]	63	Left Labium 9 cm	6 month history of pruritus treated w/corticosteroid cream. 5 cm inguinal LN metastases.	Initial: radical vulvectomy + radiotherapy	No follow up
Winer et al. 2012 [11]	69	Right inguinal 3–4 cm	Patient noted Inguinal lesion.	Initial: Surgical excision Future plans for adjuvant chemotherapy + radiotherapy	No follow up

## Discussion

The overall histopathological picture of vulvar MCC is fairly consistent with typical MCC. Histological evaluation remains the primary diagnostic modality, including a hematoxylin and eosin section along with an appropriate immunopanel. National Comprehensive Cancer Network guidelines for general MCC [7] recommend immunopanels to include CK20 and TTF-1. Most low-molecular-weight cytokeratin markers and CK20 will be positive in a perinuclear dot-like pattern, while CK7 and TTF-1 (immunoreactive in >80% of small cell lung cancers) are typically negative [7]. Neuroendocrine markers are recommended in only equivocal cases. Of the presently reviewed vulvar MCC cases, 76.5% (*n* = 13) of cases were evaluated using neuroendocrine markers, with NSE as the most commonly used (*n* = 7). While 76.5% (*n* = 13) of cases also included some sort of cytokeratin staining, only five cases were stained for CK20 and two cases were stained for CK7 (with 80% and 50% of cases positive, respectively). Histopathological workup of vulvar MCC appears to consistently include both neuroendocrine and cytokeratin markers.

A study histopathologically evaluating 21 cases [28] demonstrated MCC to express B cell lineage markers, including terminal deoxynucleotidyl transferase (TdT) and the paired box gene 5 (PAX 5). Additionally, most of the MCCs evaluated in this study expressed one or more immunoglobulin subclasses as well as kappa or lambda chains. The TdT and PAX5 coexpression is suggestive of a pro/pre- or pre-B cell origin for MCC, rather than postmitotic Merkel cells in select tumors. This disparity may aid in understanding why Merkel cell polyoma viral infection is not present in all cases. Additionally, this may have implications for therapy. Subclassification of MCC tumors by immunophenotype could create a paradigm of individualized treatment dictated by cellular origin (i.e. pre-B cell-derived tumors versus postmitotic Merkel cell tumors). However, further investigation is required to substantiate this model of MCC origin. Additionally, extensive clinical trials would be required to validate treatment regimens based on origin.

Surgical excision is the first line approach to primary MCC tumors. All reports received vulvectomy or wide local excision with 2 cm margins. National Comprehensive Cancer Network guidelines for general MCC [7] recommend sentinel lymph node biopsy followed by surgical removal using wide excision with 1–2 cm margins. Removal to investing fascia of muscle or pericranium is recommended, when clinically feasible. Additionally, physicians may consider techniques that allow more exhaustive histologic margin assessment, such as Mohs technique, modified Mohs with permanent sections for final margin assessment, or complete circumferential and peripheral deep margin assessment. No cases of vulvar MCC reported more exhaustive margin assessment such as Mohs techniques. Considering the high recurrence rate and the limits in accessibility for excision of vulvar MCC, Mohs techniques could be of potential value in the management of this condition. Such technique could improve margin control and possibly increase tissue preservation. A multi-institutional retrospective study [29] of 240 MCC cases not limited by anatomic location reported use of Mohs micrographic surgery in 13.8% of patients, most commonly with stage I disease. While overall survival of stage I/II patients did not differ with use of Mohs versus wide excision, recurrence rates and tissue preservation in these cohorts were not compared. Further evaluation of the utility of Mohs technique in vulvar MCC is warranted.

The high recurrence rate of MCC in spite of the emphasis on wide local excision and margin clearance suggests surgical management of this condition to be inadequate. In light of this poor clinical response to surgical excision, further development of medical therapies is paramount. Medical management in the available published cases was limited to cisplatin and etoposide combination therapy. With current medical management consisting only of cytotoxic chemotherapy and radiation (which causes many adverse side effects and is not mechanism-based, disease-specific therapy) there is a need for more effective and targeted treatment agents. Newer developed agents, such as TKI’s, show encouraging efficacy in other cancers and in some case reports when used for MCC. Additionally they have low toxicity and lack immune suppression due to the nature of their targeting aberrantly expressed genes commonly mutated in human cancers. A case report of metastatic MCC in a 69-year-old female demonstrated partial response to pazopanib, a tyrosine kinase inhibitor (TKI) [30]. Multiple clinical trials are underway investigating the efficacy of TKIs in MCC. These include MLN0128 (mTOR, NCT02514824), cabozantinib (c-Met and VEGFR2, NCT02036476), imatinib (NCT00068783), temsirolimus (mTOR, NCT01155258), and everolimus and vatalnib combination therapy (NCT00655655). Other biologicals are also under investigation for treatment of patients with MCC, including adjuvant ipilimumab (NCT02196961), avelumab (NCT02155647), tremelimumab and durvalumab combination therapy (NCT02643303). The age of biological and targeted therapy is rapidly changing clinical oncology, as a whole, and is promising for treatment of advanced MCC.

## Conclusion

Merkel cell carcinoma affecting the vulva is a rare and aggressive neoplasm that presents as a firm, mobile mass at a mean age of 59.6 years. Pain, ulceration, edema, and erythema may also be present. The lesion is histopathologically consistent with MCC, appearing as small hyperchromatic cells with high nucleus to cytoplasm ratio distributed in nested, trabecular patterns. Electron microscopy demonstrates cells with dense core granules and intermediate filaments. Neuroendocrine immunostain markers aid in histopathological evaluation, especially chromogranin, synaptophysin, and neuron-specific enolase. Additionally, cytokeratin stains are commonly immunoreactive, including pancytokeratin stains and CAM5.2, which will generally demonstrate a perinuclear dot or cytoplasmic granularity. Surgical management is the primary treatment modality, and adjuvant radiotherapy may be considered. However, recurrence and tumor progression are very common problems. Metastatic disease may be managed with cisplatin and etoposide combination therapy. This condition has high mortality (47.0% of 17 cases) at a mean follow-up of 7.8 months (range, 0.6–16 months) after first surgical operation. Continued investigation of targeted therapy is warranted for improved treatment in this highly aggressive disease.

## Abbreviations

AJCC: American Joint Commission of Cancer
CK20: Cytokeratin 20
MCC: Merkel cell carcinoma
TTF-1: Thyroid transcription factor-1
